# Rare-Earth-Zirconate Porous High-Entropy Ceramics with Unique Pore Structures for Thermal Insulating Applications

**DOI:** 10.3390/ma16083040

**Published:** 2023-04-12

**Authors:** Hengchang Wang, Jie Xu, Jiatong Zhu, Xuanyu Meng, Lang Lin, Ping Zhang, Feng Gao

**Affiliations:** 1 MIIT Key Laboratory of Radiation Detection Materials and Devices, State Key Laboratory of Solidification Processing, School of Materials Science and Engineering, Northwestern Polytechnical University, Xi’an 710072, China; 2 NPU-QMUL Joint Research Institute of Advanced Materials and Structure, Northwestern Polytechnical University, Xi’an 710072, China

**Keywords:** rare-earth-zirconate, porous high-entropy ceramics, thermal conductivity, gel-casting

## Abstract

Porous high-entropy ceramics are a new alternative material for thermal insulation. Their better stability and low thermal conductivity are due to lattice distortion and unique pore structures. In this work, rare-earth-zirconate ((La_0.25_Eu_0.25_Gd_0.25_Yb_0.25_)_2_(Zr_0.75_Ce_0.25_)_2_O_7_) porous high-entropy ceramics were fabricated by a tert-butyl alcohol (TBA)-based gel-casting method. The regulation of pore structures was realized through changing different initial solid loadings. The XRD, HRTEM, and SAED results showed that the porous high-entropy ceramics had a single fluorite phase without impurity phases, exhibiting high porosity (67.1–81.5%), relatively high compressive strength (1.02–6.45 MPa) and low thermal conductivity (0.0642–0.1213 W/(m·K)) at room temperature. Porous high-entropy ceramics with 81.5% porosity demonstrated excellent thermal properties, showing a thermal conductivity of 0.0642 W/(m·K) at room temperature and 0.1467 W/(m·K) at 1200 °C. The unique pore structure with a micron size contributed to their excellent thermal insulating performance. The present work provides the prospect that rare-earth-zirconate porous high-entropy ceramics with tailored pore structures are expected to be thermal insulation materials.

## 1. Introduction

Porous ceramics are often excellent materials for applications due to their low thermal conductivity [1,2,3,4]. The ultra-low thermal conductivity of porous ceramics is a primary consideration for high-temperature applications, in addition to a highly stable structure [5,6]. Great attention has been focused on low thermal conductivity ceramic systems, such as alumina, mullite, yttrium silicate and yttria-stabilized zirconia, which are used as insulating materials [7,8,9,10]. However, their further application at high temperatures is limited due to their lower structural stability and relatively high thermal conductivity. Hence, the development of new thermally insulating materials is necessary to cope with the high-temperature applications.

Rare-earth-zirconates exhibit low thermal conductivity and good mechanical properties at room temperature, suggesting the potential of rare-earth-zirconates for applications as thermal insulation materials [11,12]. In recent years, due to better stability, enhanced mechanical properties and countless possibilities in composition design, high-entropy ceramics are already attracting a lot of attention [13,14,15]. Many studies have shown that the much lower thermal conductivity of high-entropy ceramics is due to enhanced phonon scattering by the majority ion (>4) at the A sites of rare-earth-zirconates. Zhao et al. prepared (La_0.2_Ce_0.2_Nd_0.2_Sm_0.2_Eu_0.2_)_2_Zr_2_O_7_ with the same crystal structure as La_2_Zr_2_O_7_ by a solid-phase method, tested it at room temperature and found that very low values of thermal conductivity occur in high-entropy ceramics; this value is 0.76 W/(m·K) [16]. Liu et al. fabricated (La_0.2_Nd_0.2_Sm_0.2_Gd_0.2_Yb_0.2_)_2_Zr_2_O_7_ that exhibited a thermal conductivity of 1.72 W/(m·K) [17]. Furthermore, by introducing the structure of the fluorite phase, high-entropy ceramics can possess good hardness. Zhu et al. prepared (La_0.2_Nd_0.2_Sm_0.2_Gd_0.2_Yb_0.2_)_2_Zr_2_O_7_ by doping small-radius atoms (Yb) into A sites, which had a dual phase and a high hardness of 10.96 ± 0.26 GPa [18]. In addition, He et al. introduced more ions into the B sites of rare-earth-zirconates and fabricated RE_2_(Ce_0.2_Zr_0.2_Hf_0.2_Sn_0.2_Ti_0.2_)_2_O_7_ (RE are the rare earth elements, RE = Y, Ho, Er, Yb), which exhibited excellent hardness (13.5–15.7 GPa) and another excellent mechanical property, fracture toughness (1.14–1.27 MPa·m^0.5^) [19]. Zhu et al. reported that the order–disorder transition increased the scattering coefficient, which not only affected the thermal properties of A_2_B_2_O_7_ high-entropy ceramics, but the mechanical properties were also altered. The lattice thermal conductivity was further reduced to (1.52 to 1.61 W/(m·K) at 100 °C) [20].

To further improve thermal performance, it is attractive to combine the advantages of high-entropy ceramics and porous ceramics by introducing pore structures into high-entropy ceramics [21,22]. Liu et al. prepared (La_0.2_Nd_0.2_Sm_0.2_Gd_0.2_Yb_0.2_)_2_Zr_2_O_7_ porous ceramics by a foam-gel casting-freeze drying method, which had excellent and relatively low thermal conductivity (0.072 ± 0.001 W/(m·K)). The high-entropy porous ceramics with high porosity (93.3%) also possessed good and relatively high compressive strength (1.20 MPa) [23]. Meng et al. used a particle-stabilized foaming method to prepare pyrochlore-fluorite dual-phase porous ceramics (La_0.2_Nd_0.2_Sm_0.2_Gd_0.2_Yb_0.2_)_2_Zr_2_O_7_. A thermal conductivity of 0.045 W/(m·K) was found in dual-phase high-entropy ceramics, while these ceramics had a high porosity of 96.89% [24]. However, the shortcoming of the abovementioned method is that the pore sizes prepared are widely distributed, ranging from 20 to 100 μm.

Pore structures can be effectively added to ceramics by a variety of reported methods, such as particle-stabilized foaming [25,26], polymeric sponge impregnation [27], sol-gel [28], and freeze-drying [29]. The preparation methods of porous ceramics have a large influence on the final pore structure, which can result in different pore size distributions. In particular, when the pore size is less than the millimeter level, there is no convection existing in the small pores, which can effectively reduce convective thermal conductivity. Furthermore, the molecular thermal motion of air can be effectively restricted by pores of 1–5 μm in size, which also scatter the infrared radiation that contributes to the primary thermal conductivity at temperatures between 800 and 1500 °C [30,31]. As a method for preparing high-porosity ceramics, gel casting attracts widespread attention because the pore distributions of 1–10 μm in size can be successfully fabricated after drying and sintering [32,33]. In other ways, high porosity can be achieved with the suitable organic solvent environment provided by tert-butyl alcohol (TBA), as TBA has a high saturated vapor pressure and a low surface tension [34]. Therefore, the low shrinkage and good integrity of the gel network in green bodies can be easily achieved by evaporation during the drying process [35,36].

High-entropy ceramics with a composition of (La_0.25_Eu_0.25_Gd_0.25_Yb_0.25_)_2_(Zr_0.75_Ce_0.25_)_2_O_7_, abbreviated as (4RE_0.25_)_2_(Zr_0.75_Ce_0.25_)_2_O_7_, were shown to have low thermal conductivity and relatively high mechanical properties in our previous work [20]. In this work, a gel-casting method was used to prepare (4RE_0.25_)_2_(Zr_0.75_Ce_0.25_)_2_O_7_ porous high-entropy ceramics, and the method used tert-butyl alcohol (TBA) as a solvent. The regulation of the pore structure was realized through varying different initial solid loadings. The microstructure, thermal and mechanical properties of porous high-entropy ceramics were investigated.

## 2. Experimental Procedures

### 2.1. Powder Synthesis

Powders with a composition of (La_0.25_Eu_0.25_Gd_0.25_Yb_0.25_)_2_(Zr_0.75_Ce_0.25_)_2_O_7_ were synthesized by calcination under air atmosphere. The commercially available nanoscale powders RE_2_O_3_ (RE = La, Eu, Gd, Yb > 99.9%, Shanghai Naiou Nanotechnology Co., Ltd. (Shanghai, China)), ZrO_2_ (>99.9%, Shanghai Naiou Nanotechnology Co., Ltd. (Shanghai, China)) and CeO_2_ (>99.9%, Shanghai Naiou Nanotechnology Co., Ltd. (Shanghai, China)) were used as the starting materials. A molar ratio of 1:1:1:1:6:2 was used to weigh the powders, and the homogeneous mixtures were prepared after blending for 24 h. At 1200 °C, the mixture powders were transformed into the high-entropy powders after 3 h. The high-entropy powders were ground in ethanol for 24 h to avoid agglomeration and to increase particle size uniformity.

### 2.2. Fabrication

A clear and homogeneous mixed organic solution was prepared by adding the monomer and crosslinker to tert-butyl alcohol and stirring for 30 min. The premixed solution contained acrylamide (AM, C_2_H_3_CONH_2_) and N,N′-methylenebisacrylamide (MBAM, (C_2_H_3_CONH)_2_CH_2_); it had a weight ratio of AM:MBAM:TBA = 14:0.27:60. Then, 5–15 vol% prepared high-entropy powder and 0.05 wt.% polyacrylamide (the value of the organic mass is 0.05% of the mass of the high-entropy powder) were added to the premixed solution. Slurries were milled (300 rpm) for 12 h. The gelation reaction required initiators and catalysts, including N,N,N′,N,-tetramethylethylenediamine (TEMED, C_6_H_16_N_2_) and ammonium persulfate (APS, (NH_4_)_2_S_2_O_8_), respectively. Before casting, 1 wt.% of the catalyst should first be added to the slurry and mixed well, and then 15 wt.% of the initiator should be added. The mass of both is calculated according to the slurry mass. Then, the slurry was stirred for a few minutes and cast into molds. The gelation of the slurry should be carried out at 40 °C. The TBA and water in the wet green body were removed after drying for 24 h. After careful transfer to the chamber furnace, the dried green body was exposed in the air at 620 °C for 1 h to remove the polyacrylamide (PAM). Porous high-entropy ceramic was prepared at 1500 °C for 2 h.

### 2.3. Characterization

The microstructures of pre-sintered powders, the green body and porous ceramics were imaged by scanning electron microscopy (Helios G4 C, FEI, Hillsboro, OR, USA). The XRD patterns of the crystal structure were identified through X-ray diffraction (D8 DISCOVER A25, Bruker, Billerica, MA, USA). The sample was crushed, ground to a powder and well dispersed in alcohol and investigated by transmission electron microscopy (Talos F200X, Thermo Scientific, Waltham, MA, USA). A rheometer (Kinexus Pro, Malvern Instruments, Malvern, UK) was used to determine the rheological properties of liquid samples. Oxidation processes from room temperature to 800 °C of organic polymers in the green body were identified with a differential scanning calorimeter (DSC214, NETZSCH, Weimar, Germany), and the heating speed of the test apparatus is 10 °C/min in air. The bonding information was obtained by Raman spectroscopy (Renishaw Invia, Renishaw, Wotton-under-Edge, UK) using a pot size of 1 μm and a laser with a wavelength of 514 nm generated by an argon ion laser. The compressive strength of the bulk specimen was identified by a universal testing machine (GNT100, NCS Testing Technology Co., Ltd., Beijing, China), and 0.5 mm/min of the crosshead speed was used for a sample of 15 × 15 × 15 mm in size. The measurement of porosity was carried out using Archimedes’ method. At least three specimens were measured to obtain the average results for each parameter. The pore size distribution reflected the microstructure of ceramics. The results were investigated on a high-performance automatic mercury porosimeter (AutoPore Iv 9510, Micromeritics, Norcross, GA, USA). The thermal conductivity was measured using the laser method (LFA-1000, Linseis, Robbinsville Twp, NJ, USA) and a thermal conductivity detector for samples of a size of ⌀12.7 × 2 mm. A thermal infrared imaging system (FOTRIC 246M, M50, FOTRIC, Shanghai, China) was used to detect the infrared thermal management behavior of the porous ceramic samples.

## 3. Results and Discussion

**Figure 1:**
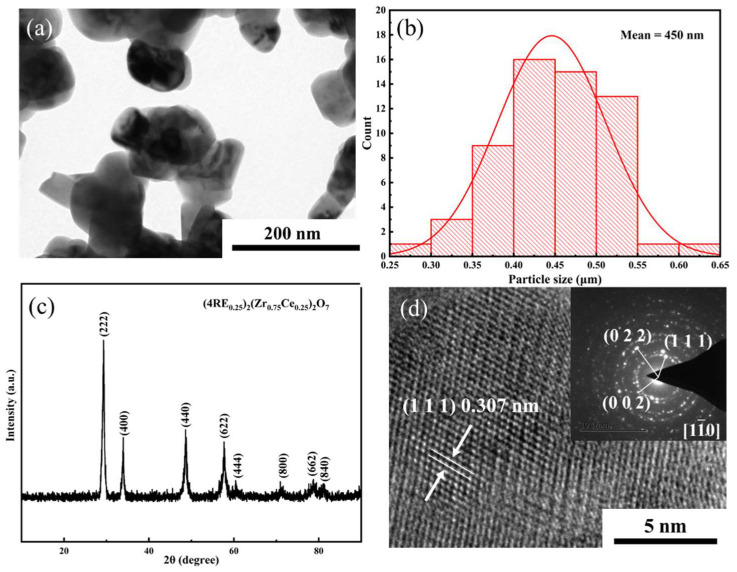
As-prepared (4RE_0.25_)_2_(Zr_0.75_Ce_0.25_)_2_O_7_ powders: (**a**) TEM image, (**b**) particle size distribution, (**c**) XRD pattern, (**d**) HRTEM image and SAED pattern with the zone axis [1$\bar{1}$0].

Figure 1b shows the particle size distribution of the (4RE_0.25_)_2_(Zr_0.75_Ce_0.25_)_2_O_7_ powders. The results illustrate a highly homogeneous size distribution of particles with a size of approximately 450 nm. As the starting powders (RE_2_O_3_, CeO_2_ powder and ZrO_2_ powder) are nanoscale (30–50 nm), Figure 1a shows that the calcined particles were submicrometric in size. High sintering activity that contributes to lowering the calcination temperature was considered in the powder. Figure 1c presents the XRD pattern of the high-entropy powders. The result indicates the single fluorite phase of (4RE_0.25_)_2_(Zr_0.75_Ce_0.25_)_2_O_7_. Owing to the larger ions (Ce^4+^) doped on B sites, the value of r_A3+_/r_B4+_ is smaller than 1.47, which is the critical value of order–disorder transition in A_2_B_2_O_7_ high-entropy ceramics [20]. This means that the component of (4RE_0.25_)_2_(Zr_0.75_Ce_0.25_)_2_O_7_ is far from the order–disorder transition phase boundary; therefore, the high-entropy powder has a single fluorite phase. To further illustrate the crystal structure of the high-entropy powder, HRTEM and SAED images are presented in Figure 1d. The HRTEM image suggests a 0.307 nm lattice spacing, corresponding to the (111) crystal plane. The lattice fringes illustrate the existence of the crystal phase in the powder after 1200 °C for 3 h, but the diffraction rings in the SAED map along the axis of the [1$\bar{1}$0] region indicate the formation of polycrystals in the high-entropy powder, which is in agreement with the XRD result.

**Figure 2:**
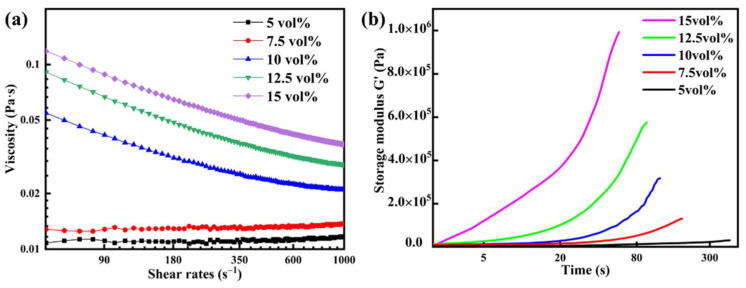
(**a**) Viscosity of premixed slurries with different solid loadings, (**b**) the storage modulus G′ in the gelling process with the slurry at different solid loadings (at 40 °C).

Slurries with different solid loadings are used for viscosity testing; the results are shown in Figure 2a for shear rate in the range 1–1000 s^−1^. The viscosity of the slurry increases from 1.08 × 10^−2^ to 3.15 × 10^−1^ Pa·s at 10 s^−1^ due to the increasing interaction force between particles with increasing solid loadings [37]. Shear rates increase slowly and uniformly from 10 to 1000 s^−1^. After testing, the apparent shear thinning behavior was found to occur in slurries with higher solid loading (10–15 vol%). However, the slurries with lower solid loadings (5–7.5 vol%) have almost no influence on the viscosity, indicating that the slurries have Newtonian fluid properties. Hence, the low viscosity is propitious to controlling the homogeneity of slurries for subsequent casting processes. Figure 2b shows the storage modulus (G′) for the slurries that had different solid loadings. At the beginning, the storage modulus of the slurries is less than 10,000 Pa, indicating the features of the original liquid. The drastic polymerization of AM results in a rapid increase in the storage modulus. The higher solid loading has a shorter polymerization time and a larger storage modulus. The distance of particles in the slurries decreases with the increasing solid loading, which indicates less time to complete the polymerization process. Meanwhile, the immobilization of the ceramic particles results in a large storage modulus.

**Figure 3:**
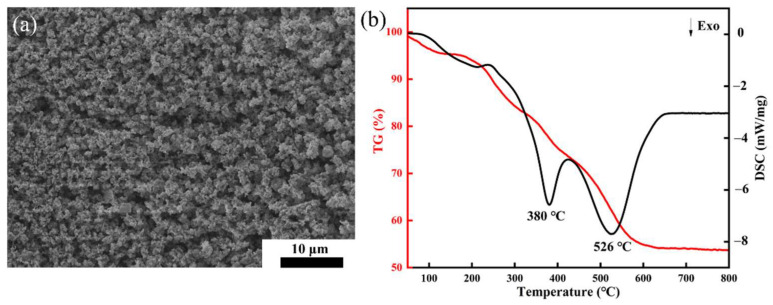
(**a**) SEM of the green body (5 vol% solid loading), (**b**) TG-DSC curves of the green body (5 vol% solid loading).

The catalyst and initiator are added to the slurries, and the ceramic particles accumulate with the condensation of AM and the connection of PAM. During the drying process at 40 °C, the solidification of PAM further expands, and green bodies are obtained after the complete evaporation of TBA. Figure 3a presents the green body’s microstructure with a 5 vol% solid loading. Open pores of several hundred nanometers to a few micrometers in size can be observed in the magnified view of the microstructure. Loose porous microstructures are formed by particle stacking. The connection of ceramic particles is dependent on the three-dimensional network of PAM and van der Waals forces.

The TG-DSC curves of the green body after gelation prepared from the 5 vol% solid loading slurry are presented in Figure 3b. When the temperature increases from 25 °C to 630 °C, the weight decreases to 54%, and the reason is mainly the elimination of PAM. First, the volatilization of water adsorbed by PAM and the residual solvent are removed at 100 °C. Then, PAM decomposes and oxidizes slowly at 120 °C, with gas emission. As the temperature increases, the polymer starts to break chains, and there is a distinct exothermic peak at 380 °C. Finally, the degradation and carbonization of small molecules begin at 430 °C with the release of carbon dioxide, which is the main reason for the exothermic peak at 526 °C. Raising the temperature further, the TG curve no longer changes. The weight loss ends at 630 °C, while the organics are eliminated. In the debinding process, the heating rate was 0.1 °C/min, and the temperature should be held at 380 °C, 550 °C and 620 °C for 60 min, after which the green bodies were obtained.

**Figure 4:**
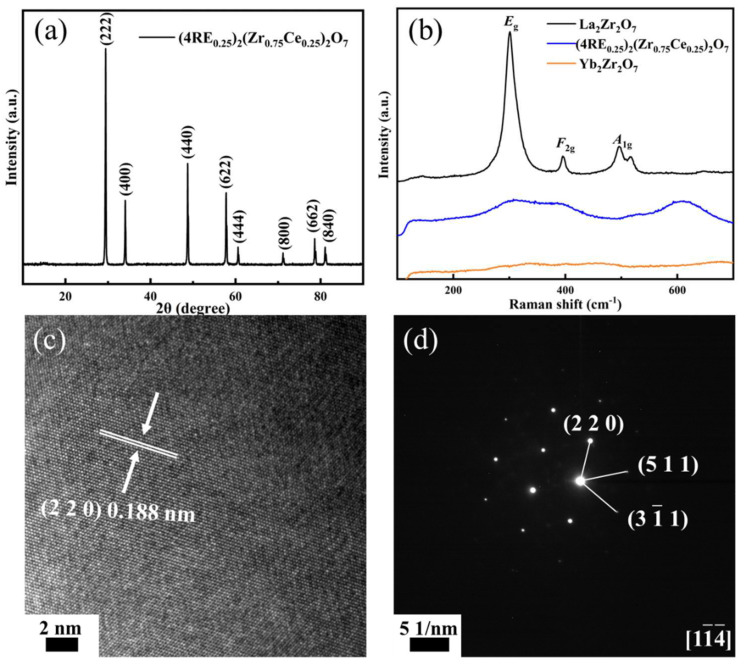
(4RE_0.25_)_2_(Zr_0.75_Ce_0.25_)_2_O_7_ porous high-entropy ceramics: (**a**) XRD pattern, (**b**) Raman spectra, (**c**) HRTEM image, (**d**) SAED pattern with the zone axis [1$\bar{1}$$\bar{4}$].

Figure 4a shows the XRD pattern of (4RE_0.25_)_2_(Zr_0.75_Ce_0.25_)_2_O_7_ porous high-entropy ceramics. After sintering for 2 h at 1500 °C, the porous high-entropy ceramics showed a single phase with a fluorite structure, and no impurity was exhibited. Because the doping content of Ce^4+^ reaches 0.25 (r_A3+_/r_B4+_ = 1.407), the ceramic component deviates from the order–disorder transition boundary and tends to form fluorite. Raman spectroscopy provides a precise expression of information on structural variations. As shown in Figure 4b, La_2_Zr_2_O_7_ exhibits the three typical Raman active vibrational modes of the pyrochlore structure: *E*_g_(O1-RE-O1), *F*_2g_(B-O1) and *A*_1g_(A-O1/O2), showing sharp peaks. In contrast, the cations and anions in the fluorite structure of Yb_2_Zr_2_O_7_ are in disordered sites. There is no obvious active vibrational mode at the corresponding Raman shift, showing a broad and weak curve [18]. The Raman spectra of the samples exhibit low Raman activity that can be attributed to the disordered structure of the high-entropy ceramics. Figure 4c,d show the HRTEM and SAED images that can further determine the structure of the high-entropy ceramics. The porous high-entropy ceramic after sintering at 1500 °C exhibits sharp lattice fringes and good crystallinity. The HRTEM image suggests a lattice spacing of 0.188 nm, corresponding to the (220) plane. The SAED plot along the axis of the [1$\bar{1}$$\bar{4}$] region discloses that the high-entropy ceramics have a single fluorite structure, which is consistent with the XRD pattern. Therefore, the diffraction pattern proves the single fluorite structure of ceramics.

**Figure 5:**
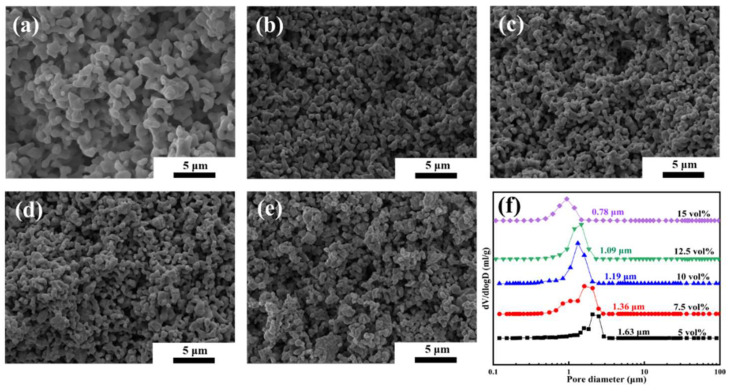
(**a**–**e**) Microstructures of (4RE_0.25_)_2_(Zr_0.75_Ce_0.25_)_2_O_7_ porous high-entropy ceramics with different solid loadings (5–15 vol%), (**f**) Pore size distribution of ceramics with different solid loadings.

The microstructure images are shown in Figure 5a–e. A uniform and irregular pore opening structure is observed in all samples. With the evaporation of TBA in different directions and the 3D stacked network structure via PAM, the interconnected porous structure forms after diffusion and growth between particles at high-temperature sintering. These ceramics are integral and have no cracks. In addition, the size of the ceramic grains is approximately 1 μm. The ceramic particles easily pile up into clumps at 15 vol% solid loading. The distance between particles is so short that the closed cell structure tends to appear.

**Figure 6:**
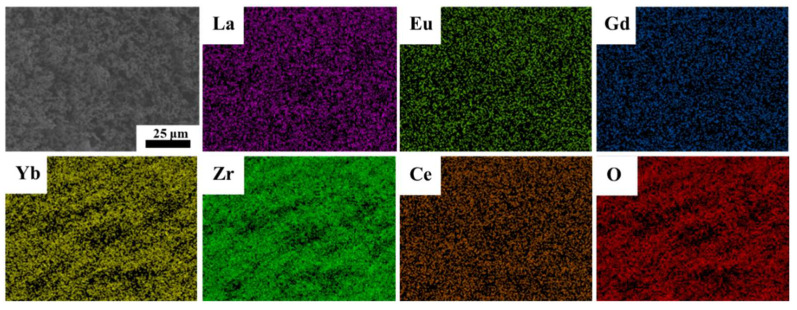
Elements distribution of (4RE_0.25_)_2_(Zr_0.75_Ce_0.25_)_2_O_7_ porous high-entropy ceramics prepared from 5 vol% suspension.

The average pore size distribution is presented in Figure 5f. The average pore size of porous ceramics with different solid loadings is approximately 1 μm and has a single peak feature, which indicates that there is no extensive particle agglomeration. For the porous ceramics, the average pore size decreases from 1.63 μm to 1.36 μm when the solid loadings change from 5 vol% to 7.5 vol%. However, some pore distribution is present at the micronanoscale when the solid loading is 7.5 vol%, which broadens the peak of the pore distribution. By further increasing the solid loading to 10 vol%, the micronanoscale pores disappear, and the average pore size decreases to 1.19 μm. At high solid loadings (12.5–15 vol%), the peak of the pore distribution becomes wide, resulting in a smaller average pore size. The EDS mapping of porous high-entropy ceramics prepared from 5 vol% suspension is shown in Figure 6. The EDS mapping suggested a uniform distribution of these rare earth elements with no segregation. In summary, porous high-entropy ceramics with micrometer opening structures and high porosity are fabricated by the tert-butyl alcohol-based gel-casting method.

**Figure 7:**
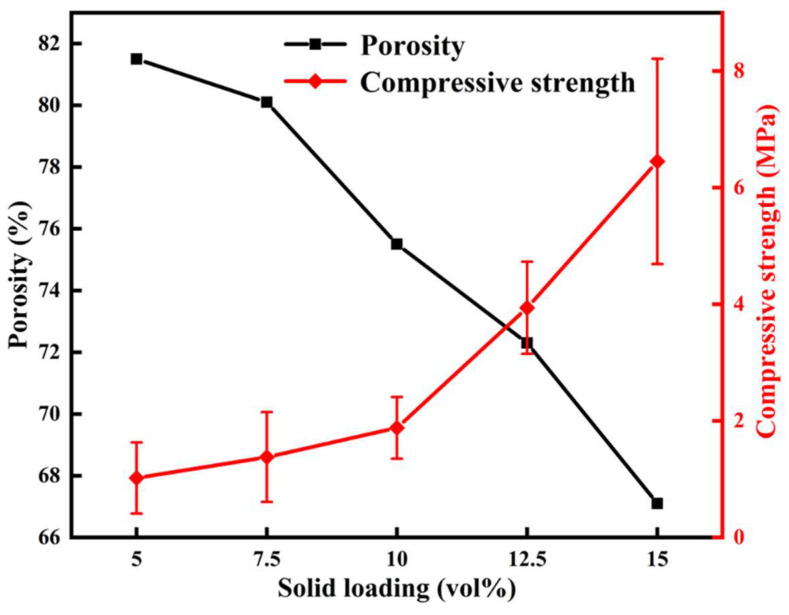
Porosity and compressive strength of (4RE_0.25_)_2_(Zr_0.75_Ce_0.25_)_2_O_7_ porous high-entropy ceramics with different solid loadings.

Figure 7 shows the porosity and compressive strength of (4RE_0.25_)_2_(Zr_0.75_Ce_0.25_)_2_O_7_ porous high-entropy ceramics with different solid loadings. The porous high-entropy ceramics have relatively high porosity, decreasing from 81.5% to 67.1% when the solid loading increases from 5 vol% to 15 vol%. Simultaneously, the decreasing pore size and the increasing particle number cause low porosity. Compared to the reported single-component La_2_Zr_2_O_7_ system (84.2–67.9%) [38], the porous high-entropy ceramics have lower porosity. Because of the doping of Ce^4+^ and high-entropy ions in the A site at the same sintering temperature, the optimum sintering temperature decreases, and the shrinkage increases. From the Rice model, when the porosity (P) is higher, the compressive strength is lower [39]. Due to the reduced particle distribution of ceramics, the number of particle-to-particle sintered neck structures that provide primary strength decreases, and the connections are not tight enough. The compressive strength increases from 1.02 ± 0.61 MPa to 6.45 ± 1.76 MPa, which is lower than that of La_2_Zr_2_O_7_ (1.56–7.89 MPa) fabricated by the tert-butyl alcohol-based gel-casting method [38].

**Figure 8:**
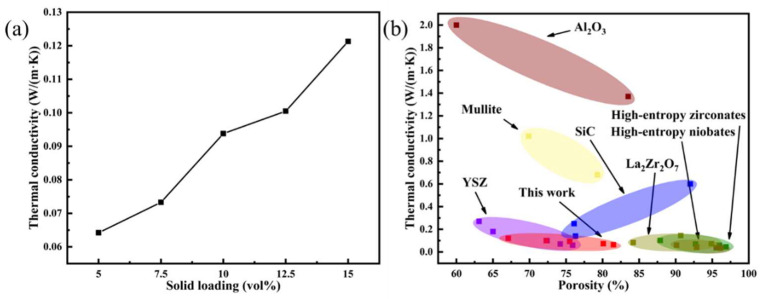
(**a**) Thermal conductivity of (4RE_0.25_)_2_(Zr_0.75_Ce_0.25_)_2_O_7_ porous high-entropy ceramics with different solid loadings, (**b**) thermal conductivity of typical porous thermal insulating ceramics.

The thermal conductivity at room temperature of porous high-entropy ceramics is presented in Figure 8a. The results show that the thermal conductivity increases with increasing solids content. Porous ceramics with 5 vol% solid loading have the lowest room temperature thermal conductivity (0.0642 W/(m·K)). The direct reason for the lower thermal conductivity is the higher-porosity ceramics. The phase of air has a low thermal conductivity, which means that uniformly dispersed pores in porous ceramics correspond to good adiabatic phases. In addition, high-entropy ceramic particles also have low thermal conductivity. Hence, the porous ceramics possess lower thermal conductivity.

Figure 8b and Table 1 show the comparison of (4RE_0.25_)_2_(Zr_0.75_Ce_0.25_)_2_O_7_ porous high-entropy ceramics and common porous thermal insulation system materials. Due to the low intrinsic thermal conductivity of the selected high-entropy system, the porous high-entropy ceramics have lower room temperature thermal conductivity than the single-component La_2_Zr_2_O_7_ porous ceramic prepared by gel casting [38] (67.9–84.2%, 0.083–0.207 W/(m·K)) and the particle stabilized foaming method [40] (90.7–94.9%, 0.073–0.144 W/(m·K)). The thermal conductivity of this work is close to that of porous high-entropy ceramic (La_0.2_Nd_0.2_Sm_0.2_Gd_0.2_Yb_0.2_)_2_Zr_2_O_7_ (87.99–96.89%, 0.045–0.102 W/(m·K)) by the particle stabilized foam method with similar porosity. In addition, the compressive strength of this work is higher than that of porous high-entropy ceramics (96.89%, 0.283 MPa) with high porosity prepared by the particle-stabilized foaming method [24].

However, the high-entropy rare-earth-niobate (5RE_3_NbO_7_) porous ceramics possess high porosity (90.13–96.12%). Due to the high-entropy effect and high porosity, the thermal conductivity of ceramics is low (0.0343–0.0592 W/(m·K)) [41]; yet, the compressive strength is low (0.273–0.528 MPa). Compared with the traditional thermal insulation system YSZ (63.1–75.9%, 0.06–0.27 W/(m·K)) [42], Al_2_O_3_ (60–83.51%, 1.37–2 W/(m·K)) [7,43], SiC (76.1–92%, 0.14–0.6 W/(m·K)) [44,45] and mullite (69.9–79.3%, 0.68–1.02 W/(m·K)) [46], (4RE_0.25_)_2_(Zr_0.75_Ce_0.25_)_2_O_7_ porous high-entropy ceramics have high strength, low thermal conductivity and a good prospect of thermal insulation application.

**Table 1:** Comparison of porosity and room temperature thermal conductivity of typical porous thermal insulating ceramics.

Samples	Porosity (%)	Compressive Strength (MPa)	Thermal Conductivity (W/(m·K))	Reference
YSZ(ZrO_2_-8mol% Y_2_O_3_)	72.4	9.64	0.10	[42]
	74.2	7.92	0.07	
	75.9	3.04	0.06	
(Dy_0.2_Ho_0.2_Y_0.2_Er_0.2_Yb_0.2_)_3_NbO_7_	96.13	0.273	0.0343	[41]
	95.56	0.306	0.0377	
	92.88	0.346	0.0407	
	90.13	0.64	0.0592	
Al_2_O_3_	60	/	2	[7,43]
	83.51	3.84	1.37	
SiC	76.1	/	0.25	[44,45]
	76.3	/	0.14	
	92	/	0.6	
Mullite	79.3	1.22	0.68	[46]
	69.9	5.12	1.02	
(La_0.2_Nd_0.2_Sm_0.2_Gd_0.2_Yb_0.2_)_2_Zr_2_O_7_	96.89	0.283	0.045	[24]
	96.00	0.419	0.06	
	92.76	1.232	0.07	
	87.99	4.167	0.102	
La_2_Zr_2_O_7_	94.9	1.19	0.073	[38,40]
	90.7	3.05	0.144	
	84.2	1.56	0.083	
	67.9	7.89	0.207	
(La_0.25_Eu_0.25_Gd_0.25_Yb_0.25_)_2_(Zr_0.75_Ce_0.25_)_2_O_7_	81.5	1.02	0.0642	This work
	75.5	1.88	0.0938	

**Figure 9:**
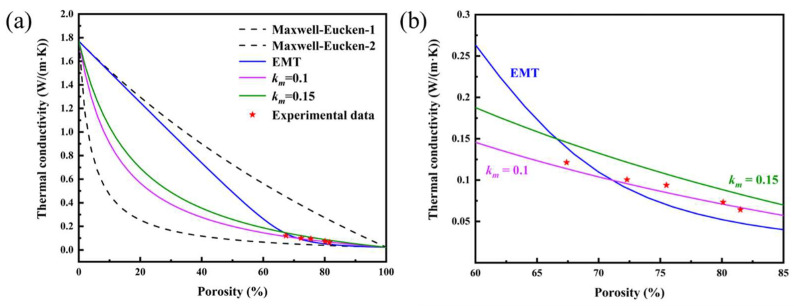
(**a**) Experimental thermal conductivity and theoretical model curves of (4RE_0.25_)_2_(Zr_0.75_Ce_0.25_)_2_O_7_ porous high-entropy ceramics with different porosities. (**b**) Magnified image of the area corresponding to the experimental data.

For the relationship between the porosity and thermal conductivity of two-phase compound materials, Hashin and Shtrikman [47] proposed the Maxwell–Eucken equation, which is represented by the following two equations:

(1) $$k_{e}=k_{1}\frac{2k_{1}+k_{2}-2\left(k_{1}-k_{2}\right)v_{2}}{2k_{1}+k_{2}+\left(k_{1}-k_{2}\right)v_{2}}$$

(2) $$k_{e}=k_{2}\frac{2k_{2}+k_{1}-2\left(k_{2}-k_{1}\right)(1-v_{2})}{2k_{2}+k_{1}+\left(k_{2}-k_{1}\right)\left(1-v_{2}\right)}$$

where *k_e_* is the composite thermal conductivity, *k*_1_ is the thermal conductivity of the dispersed phase, *k*_2_ is the thermal conductivity of the medium phase and *v*_2_ is the porosity. *k*_1_ is obtained by the laser testing of dense ceramics, and the value is 1.66 W/(m·K) [20]. *k*_2_ is equal to air in thermal conductivity. Equation (2) describes the case in which the thermal conductivity of the dispersed phase is larger than that of the continuous phase, and Equation (3) describes the inverse case. However, the curves calculated and tested in Figure 9a show that the results with the calculation of the Maxwell–Eucken equation are out of position, because the equation lacks consideration of heat transport when the dispersed or medium phase is continuous. To further revise these results, the effective medium theory model (EMT model) can be used to describe the relationship between thermal conductivity and porosity for the random distribution of the two phases, which is represented by the following formula [48]:

(3) $$\left(1-v_{2}\right)\frac{k_{1}-k_{e}}{k_{1}+2k_{e}}+v_{2}\frac{k_{2}-k_{e}}{k_{2}+2k_{e}}=0$$

However, the fitting result of the EMT model indicated that the experimental results do not completely fit the EMT theoretical model curve, which may be caused by the uneven distribution of pore size in the prepared porous ceramics. The new effective medium theory model (GM model) can further correct the effect of nonuniform pore size on thermal conductivity, which can be expressed by the following formula:

(4) $$\left(1-v_{2}\right)\frac{k_{1}-k_{e}}{k_{1}+2k_{m}}+v_{2}\frac{k_{2}-k_{e}}{k_{2}+2k_{m}}=0$$

where *k_m_* is the third medium phase with different thermal conductivities from the dispersed and continuous phases. When *k_m_* = *k*_1_ (~0.1 W/(m·K)), there are no closed pores in the dispersed phases, and the thermal conductivity of the medium is the thermal conductivity of air. When *k_m_* = *k*_2_ (0.026 W/(m·K)), the dispersed phase is equal to air in thermal conductivity. Hence, when *k_m_* is larger, the possibility of the pores participating in heat conduction and being closed pores is higher. Figure 9b presents the experimental data fitted with the predicted values from the model curve of *k_m_* = 0.1 W/(m·K). Because the sintering shrinkage is small, it is difficult to form a closed-cell structure.

**Figure 10:**
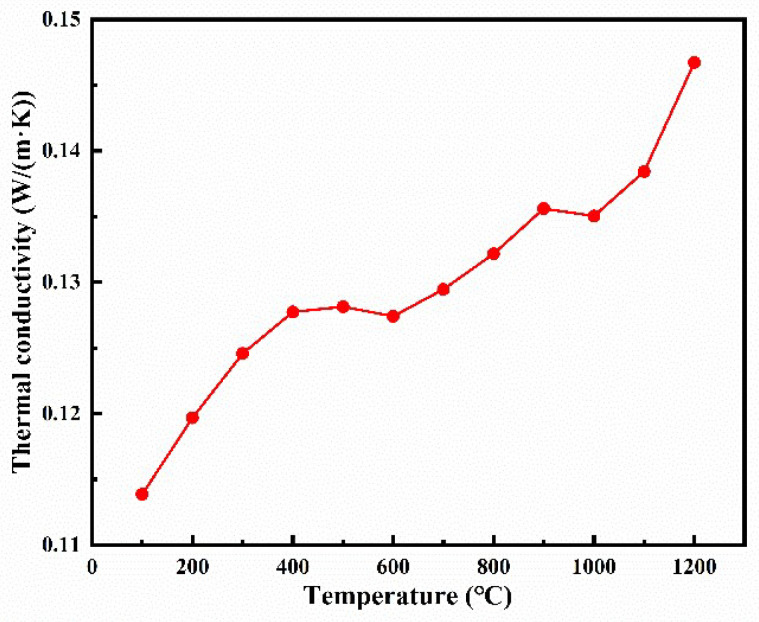
Thermal conductivity versus temperatures for the (4RE_0.25_)_2_(Zr_0.75_Ce_0.25_)_2_O_7_ ceramics.

Figure 10 illustrates the experimentally determined thermal conductivity with respect to temperature from 100 to 1200 °C. The solid loading and porosity of the sample tested are 5 vol% and 81.5%, respectively. Because the unique pore structure formed by gel casting and the phonon free path decreases gradually with increasing temperature, the thermal conductivity is still only 0.1467 W/(m·K) at 1200 °C. A rapid increase in the thermal conductivity occurs in the low-temperature range from 100 °C to 500 °C. Because the heat conduction of gas molecules intensifies when the temperature increases, the thermal conductivity of ceramics increases. The radiative thermal conductivity gradually increases in the range of 600–1200 °C. At a constant temperature, the radiative thermal conductivity is transmitted by electromagnetic waves with a certain range of wavelengths, and the wavelength corresponding to the peak can be calculated by Wayne’s shift law. The wavelength corresponding to the peak of the radiant heat transfer is 1.6–2.9 μm [30]. Porous ceramics prepared from the 5 vol% solid loading slurry possess a uniform distribution pore structure and interconnected particle structure. In addition, the random distribution of the dispersed phase and continuous phase can be determined from the results of the EMT and GM models. The size of the ceramic particles and average pore are 1–2 μm and 1.6 μm, respectively. The size of the pore structure is identical to the electromagnetic wavelength of radiant heat transfer at high temperatures. According to the Mie scattering law, evenly dispersed colloidal systems can effectively scatter electromagnetic waves that are less than or equal to the wavelength at the peak. So, it is this unique pore structure that impedes heat propagation at high temperatures, which can reduce the radiative thermal propagation [30]. It is clearly observed that the (4RE_0.25_)_2_(Zr_0.75_Ce_0.25_)_2_O_7_ porous high-entropy ceramics presented lower thermal conductivity values, which demonstrates their potential application as thermal insulation materials.

**Figure 11:**
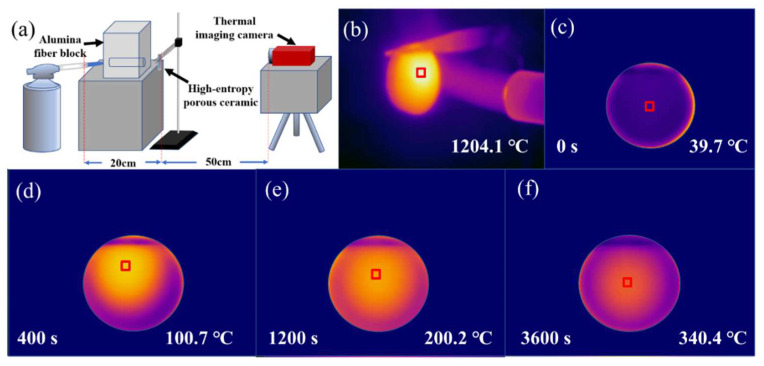
(**a**) Schematic diagram of the testing device, (**b**–**f**) Optical and infrared images of the testing sample (5 vol% solid loading, the red box represents the highest temperature zone of the sample).

Optical and infrared images are shown in Figure 11. The thickness of the testing sample is 7.08 mm. Figure 11a clearly exhibits the experimental process. The temperature of the flame can reach 1204 °C, which is presented in Figure 11b. With the alumina fiber block, the flame can be concentrated and stabilized. As shown in Figure 11c–f, the temperature of the back can reach 340.4 °C during the long testing process (1 h) before the sample has obvious cracks. This also indicates that the sample could withstand prolonged high-temperature flames. Therefore, the (4RE_0.25_)_2_(Zr_0.75_Ce_0.25_)_2_O_7_ porous high-entropy ceramics have good thermal stability and insulation performance.

## 4. Conclusions

(4RE_0.25_)_2_(Zr_0.75_Ce_0.25_)_2_O_7_ porous high-entropy ceramics are fabricated using a TBA-based gel casting method with powders of approximately 450 nm. The regulation of their pore structures is achieved through varying different initial solid loadings (from 5 to 15 vol%). All these ceramics possess a single fluorite phase, a uniform distribution of elements and even an open cell structure. The as-prepared porous high-entropy ceramics have high porosity (81.5–67.1%) and a small average pore size (0.78–1.63 μm). These pores hinder heat transfer propagation and therefore result in low room temperature thermal conductivity (0.0642–0.1213 W/(m·K)). The uniform pores also lead to moderately high compressive strength (1.02–6.45 MPa). Porous high-entropy ceramics prepared from the 5 vol% solid loading suspension have a porosity of 81.5%. High porosity leads to a thermal conductivity at 1200 °C 0.1467 W/(m·K). The results suggest that porous high-entropy ceramics have outstanding thermal insulation properties and are desirable candidates for high-temperature insulation applications.

## Data Availability

The data that support the findings of this study are available on request from the corresponding author, [xujie@nwpu.edu.cn], upon reasonable request.
